# Imeglimin prevents human endothelial cell death by inhibiting mitochondrial permeability transition without inhibiting mitochondrial respiration

**DOI:** 10.1038/cddiscovery.2015.72

**Published:** 2016-01-18

**Authors:** D Detaille, G Vial, A-L Borel, C Cottet-Rouselle, S Hallakou-Bozec, S Bolze, P Fouqueray, E Fontaine

**Affiliations:** 1 University Grenoble Alpes, LBFA, F-38000 Grenoble, France; 2 INSERM, U1055, F-38000 Grenoble, France; 3 Poxel, F-69000 Lyon, France

## Abstract

Imeglimin is the first in a new class of oral glucose-lowering agents, having recently completed its phase 2b trial. As Imeglimin did show a full prevention of β-cell apoptosis, and since angiopathy represents a major complication of diabetes, we studied Imeglimin protective effects on hyperglycemia-induced death of human endothelial cells (HMEC-1). These cells were incubated in several oxidative stress environments (exposure to high glucose and oxidizing agent *tert*-butylhydroperoxide) which led to mitochondrial permeability transition pore (PTP) opening, cytochrome *c* release and cell death. These events were fully prevented by Imeglimin treatment. This protective effect on cell death occurred without any effect on oxygen consumption rate, on lactate production and on cytosolic redox or phosphate potentials. Imeglimin also dramatically decreased reactive oxygen species production, inhibiting specifically reverse electron transfer through complex I. We conclude that Imeglimin prevents hyperglycemia-induced cell death in HMEC-1 through inhibition of PTP opening without inhibiting mitochondrial respiration nor affecting cellular energy status. Considering the high prevalence of macrovascular and microvascular complications in type 2 diabetic subjects, these results together suggest a potential benefit of Imeglimin in diabetic angiopathy.

## Introduction

Imeglimin is the first in a new tetrahydrotriazine-containing class of oral glucose-lowering agents – the Glimins – and has just completed a phase 2b clinical trial (US/EU EudraCT number 2012-004045-33). Several clinical trials evidenced Imeglimin efficacy on HbA1c as a mono and add-on therapy,^1–3^ being well tolerated. Imeglimin has effects on the liver, muscle and the pancreas,^4^ three key organs involved in type 2 diabetes pathophysiology, through suspected mechanisms involving the mitochondria and reduced oxidative stress. Imeglimin decreases hepatic glucose production and increases muscle glucose uptake.^4^ Imeglimin also demonstrated increased insulin secretion in response to glucose in diabetic patients during a hyperglycemic clamp study.^5^ Recently, it was demonstrated in high fat high sucrose diet mice that Imeglimin normalizes glucose tolerance and insulin sensitivity by protecting mitochondrial function from oxidative stress and favoring lipid oxidation in the liver.^6^ In addition to its antidiabetic effects, Imeglimin also exhibited a protective effect on β-cell apoptosis induced by various stresses (high glucose or inflammatory cytokine cocktail).^4^

Mitochondria are involved in numerous physiological processes including energy metabolism, calcium homeostasis and programmed cell death.^7–9^ Several mitochondrial proteins such as cytochrome *c* or AIF, which have no proapoptotic activity when they remain inside mitochondria, promote cell death once released into the cytosol.^10,11^ The permeability transition pore (PTP) is a Ca^2+^-sensitive mitochondrial inner membrane channel.^12,13^ Normally closed in order to allow ATP synthesis, permanent PTP opening leads to a drastic inhibition of ATP synthesis through the collapse of the proton-motive force, a dramatic increase in reactive oxygen species (ROS) production^14,15^ and a release of mitochondrial proapoptotic proteins,^16^ which results in cell death.^8^ Ca^2+^ is the most important factor for PTP opening. The amount of Ca^2+^ required to open the pore varies according to a number of factors. PTP inhibitors and PTP inducers refer to factors that increase and decrease the amount of Ca^2+^ required to induce PTP opening.^17^ Cyclosporine A (CsA) is the reference PTP inhibitor, whereas oxidative stress is recognized to favor PTP opening.^13^ In several cell types, direct or indirect inhibition of respiratory chain complex I has been shown to prevent PTP opening.^18–23^

Angiopathy represents a major complication of diabetes that determines the quality of life and life expectancy of the diabetic patients.^24^ Since Imeglimin has been shown to exhibit a protective effect on glucose-induced cell death in β-cells,^4^ the objective of this study was to establish whether Imeglimin prevents hyperglycemia-induced cell death in human endothelial cells and to clear the mechanistic basis for its antiapoptotic property. We demonstrate that Imeglimin prevents hyperglycemia-induced cell death in HMEC-1 cells by inhibiting PTP opening without inhibiting mitochondrial respiration and strongly decreases ROS specifically produced by reverse electron transport at the mitochondrial complex I level.

## Results

### Prevention of cell death by Imeglimin

**Figure 1:**
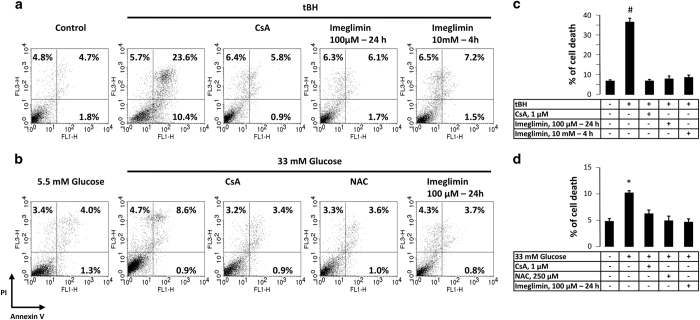
Effect of Imeglimin on tBH- or hyperglycemia-induced endothelial cell death. HMEC-1 cells incubated in the absence or presence of either 100 *μ*M Imeglimin for 24 h, 10 mM Imeglimin for 4 h or 1 *μ*M CsA for 30 min were washed with PBS before subsequent exposure to 0.5 mM tBH or vehicle (control) in FBS-free culture medium for 45 min. Cells were then washed with PBS and incubated at 37 °C for 24 h in a complete MCDB medium. Alternatively, HMEC-1 cells were cultured for 48 h in a complete MCDB medium at the indicated concentration of glucose, in the absence or presence of 1 *μ*M CsA, 250 *μ*M *N*-acetyl cysteine (NAC) or 100 *μ*M imeglimin. Cytotoxicity was evaluated by staining cells with Alexa Fluor-conjugated annexin V and PI. (**a** and **b**) Representative data. (**c** and **d**) Percentage of dead cells (i.e., cells positive for annexin V or PI) in five different experiments. Results are mean±S.E.M.; ^#^ *P*<0.01, *P<0.05 *versus* control cells, paired Student’s *t*-test.

In order to establish whether Imeglimin can prevent human endothelial cell death, HMEC-1 cells were exposed either to *tert*-butylhydroperoxide (tBH) or high concentration of glucose, two conditions known to induce PTP opening and cell death.^20^ Exposure of HMEC-1 cells to tBH led to a significant fivefold increase in cell death that was prevented by the PTP inhibitor CsA (Figures 1a–c). Preincubation with Imeglimin (10 mM for 4 h or 100 *μ*M for 24 h) also fully prevented tBH-induced cell death. When HMEC-1 cells were cultured under high glucose concentration, the percentage of dead cells almost doubled after 48 h (Figures 1b–d), and this was prevented by general antioxidant *N*-acetyl cysteine and CsA, in agreement with the proposal that hyperglycemia is responsible for an oxidative stress that in turn induces PTP opening. Imeglimin similarly prevented hyperglycemia-induced endothelial cell death as shown in Figure 1.

### Imeglimin prevents tBH- and high glucose-induced cytochrome *c* release

**Figure 2:**
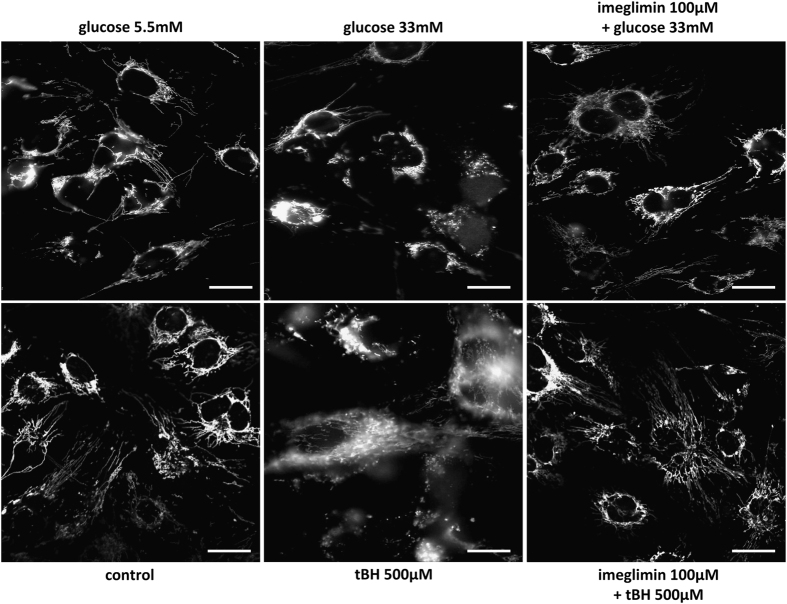
Effect of Imeglimin on cytochrome *c* distribution. HMEC-1 cells incubated in the presence or absence of Imeglimin were exposed to tBH or to hyperglycemic conditions as described in Figure 1. After 24 or 48 h (for tBH treatment and hyperglycemia, respectively), immunostaining was performed with specific anti-cytochrome *c* antibody. Representative data of five different experiments. Scale bar: 40 *μ*m.

To clear whether Imeglimin inhibits the cell death cascade before or after the release of mitochondrial proapoptotic proteins, we investigated the subcellular distribution of cytochrome *c* in stressed HMEC-1. As shown in Figure 2 (left panels), cytochrome *c* in control HMEC-1 cells was located within mitochondria. Incubation of endothelial cells in the presence of 33 mM glucose for 48 h, or 45 min exposure to 0.5 mM tBH followed by 24 h incubation in normal medium, induced a release of cytochrome *c* in the cytoplasm (i.e., a presence of cytochrome *c* outside mitochondria) in some endothelial cells (Figure 2, middle panels). Imeglimin prevented cytochrome *c* decompartmentalization (Figure 2, right panels).

### Imeglimin delays PTP opening in HMEC-1 cells

**Figure 3:**
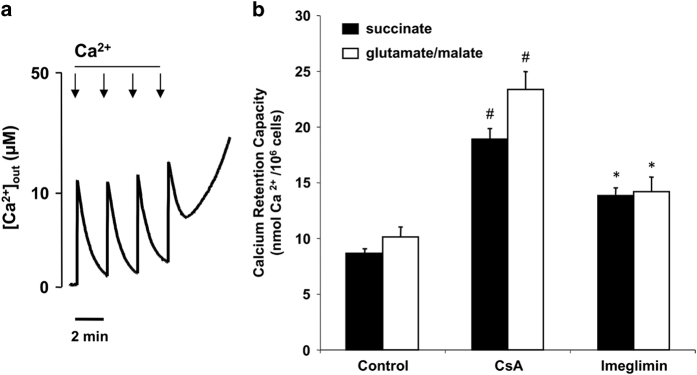
Effects of Imeglimin on the Ca^2+^ retention capacity of digitonin-permeabilized HMEC-1 cells. (**a**) The incubation medium contained 250 mM sucrose, 1 mM Pi, 10 mM Tris-MOPS, 0.25 *μ*M Calcium Green-5N, 50 *μ*g/ml digitonin and either 5 mM succinate or 5 mM glutamate plus 2.5 mM malate. The final volume was 1 ml (pH 7.35) at 25 °C. Experiments were started by the addition of 10^7^ HMEC-1 cells. Where indicated, 12.5 *μ*M Ca^2+^ pulses were added (arrows). Panel (**b**) represents cumulative data of five different experiments performed as described in the panel (**a**) after preincubation with CsA (1 *μ*M for 30 min) or Imeglimin (100 *μ*M for 24 h). Results are mean±S.E.M.; ^#^ *P*<0.01; *P<0.05 *versus* control cells, paired Student’s *t*-test.

CsA inhibited PTP opening in permeabilized HMEC-1 cells as shown by an increase in calcium retention capacity (CRC) (i.e., the amount of Ca^2+^ required to induce PTP opening) (Figure 3b). Preincubation with 100 *μ*M Imeglimin for 24 h also increased the CRC, although the effect was less pronounced than the inhibition by CsA (Figure 3b). The inhibitory effect was observed when mitochondria were energized with either complex I (glutamate/malate) or complex II (succinate) substrates.

**Figure 4:**
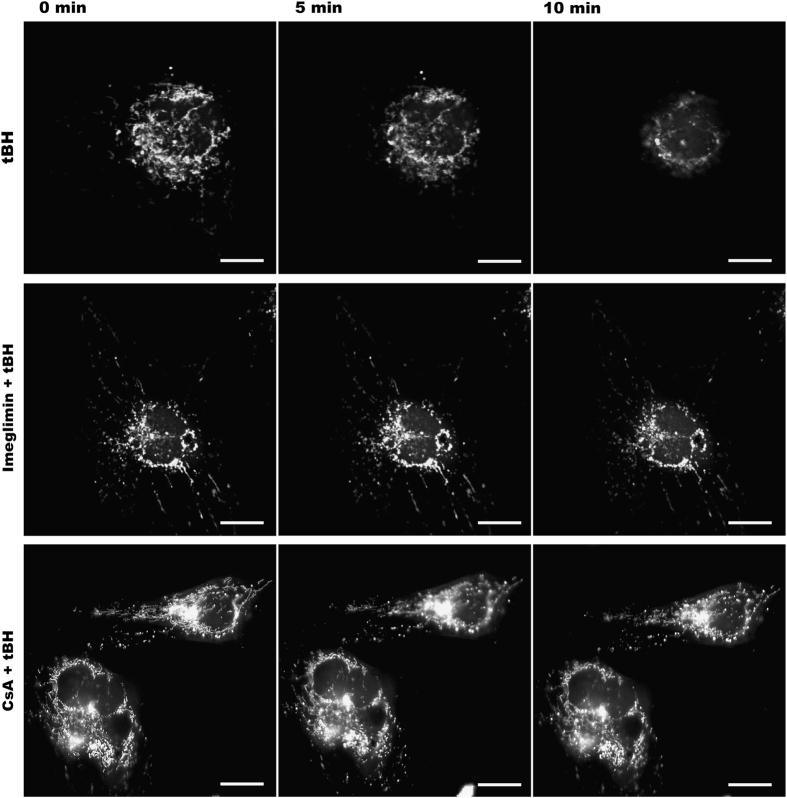
Effect of Imeglimin on tBH-induced PTP opening in intact HMEC-1 cells. HMEC-1 cells incubated in the absence or presence of either 100 *μ*M Imeglimin for 24 h or 1 *μ*M CsA for 30 min were co-loaded for 15 min with calcein plus cobalt and exposed to tBH. Images were collected every 2 min with an inverted confocal microscope, using a ×63 water immersion objective. Representative data of five different experiments. Scale bar: 40 *μ*m.

We also analyzed the effect of Imeglimin on PTP opening in intact HMEC-1 cells, which can be visualized using fluorescent compounds: calcein acetomethoxyl ester and cobalt, that do not enter mitochondria unless the PTP is open. In our experimental conditions, calcein loaded both cytosol and mitochondria but the fluorescence from cytosolic calcein was quenched by the addition of cobalt that distributes in cells but not in mitochondria. In such condition, the calcein fluorescence remains compartmentalized within mitochondria until PTP opening allows the distribution of cobalt inside mitochondria, which results in the quenching of calcein fluorescence. The addition of tBH led to calcein decompartmentalization (i.e., PTP opening), and this was delayed by CsA.^19,20^ As shown in Figure 4, Imeglimin (100 *μ*M for 24 h) also delayed tBH-induced calcein decompartmentalization in intact cells. Together, these observations demonstrate that Imeglimin is a novel PTP inhibitor.

### Imeglimin does not inhibit mitochondrial respiration

As shown in Table 1, Imeglimin did not inhibit rotenone-sensitive NADH-ubiquinone oxidoreductase activity (i.e., complex I activity). Imeglimin did not affect oxygen consumption rates in intact endothelial cells (Table 2), did not stimulate glycolysis (lactate production) and did not affect either the cytosolic redox potential (as assessed by the lactate-to-pyruvate ratio) or the phosphate potential (as assessed by the ATP/ADP ratio). In contrast, Metformin, which partly inhibits complex I (Table 1), decreased the spontaneous and stimulated (dinitrophenol (DNP)) oxygen consumption of intact cells (Table 2). Metformin increased the cytosolic redox potential and decreased the phosphate potential despite a stimulation of glycolysis (Table 2).

### Imeglimin inhibits ROS production linked to reverse electron flux through complex I

As shown in Table 3, Imeglimin did not inhibit H_2_O_2_ production when permeabilized cells were incubated in the presence of complex I substrates (glutamate/malate) either in the resting condition or when H_2_O_2_ production was increased by complex I or complex III inhibitors (rotenone and antimycin A, respectively), indicating that Imeglimin does not exhibit antioxidant activity. Permeabilized cells were then incubated in the presence of complex II substrates alone (succinate) or in combination with glutamate and malate. In this situation (i.e., when the respiratory chain is energized with complex II substrate), most of the ROS production is due to a reverse electron flux through complex I and is abolished by complex I inhibitors. As expected, rotenone decreased H_2_O_2_ production when permeabilized cells were incubated in the presence of succinate. Imeglimin dramatically decreased H_2_O_2_ production before rotenone addition, suggesting that Imeglimin inhibited reverse electron flux through complex I.

## Discussion

In this study we have shown that Imeglimin exhibits antiapoptotic properties in human endothelial cells, preventing cell death induced by oxidative stresses such as hyperglycemia and tBH. This antiapoptotic property is not restricted to human endothelial cells, since this benefit was previously reported in INS-1 cells and in rat pancreatic β-cells.^4^ We report for the first time that Imeglimin acts as a PTP inhibitor that prevents Ca^2+^-induced PTP opening in permeabilized HMEC-1 cells and tBH-induced PTP opening in intact HMEC-1 cells. We conclude that Imeglimin prevents cell death in HMEC-1 cells through inhibition of PTP opening.

Different ways to inhibit PTP opening are described in the literature. While the reference inhibitor CsA inhibits PTP opening by removing cyclophilin D from the rest of the pore,^25,26^ we have shown over the last decade that others such as rotenone and Metformin inhibit PTP opening due to complex I inhibition.^18,20–23,27^ Despite the molecular nature of the PTP remains debated, these observations led us to propose a model in which PTP opening is regulated by the molecular conformation of complex I,^23^ this latter being known to be affected by complex I inhibitors.^28–30^ In the present study, Imeglimin did not inhibit either complex I activity or oxygen consumption rates in intact HMEC cells, whereas it decreased ROS production induced by reverse electron flux through complex I. Today, the mechanism by which Imeglimin inhibits PTP opening is not known but we however suggest that Imeglimin may affect the molecular conformation of complex I, without inhibiting its activity (see below).

Mitochondria are the main source of ROS production in cells. Superoxide can be generated both at respiratory chain complexes I and III and is secondarily converted in H_2_O_2_ by the superoxide dismutase.^31^ Complex I is a reversible proton pump that can generate superoxide during forward and reverse electron flux.^32,33^ The classical complex I inhibitor rotenone increases ROS production driven by a forward electron flux, whereas it decreases ROS production driven by a reverse electron flux.^34^ The ROS production driven by a reverse electron flux is also very sensitive to the mitochondrial membrane potential, and therefore decreases when mitochondrial respiration is increased by ATP synthesis or by uncoupling.^35,36^ Theoretically, the mechanism by which Imeglimin specifically decreases the ROS production driven by a reverse electron flux would imply that Imeglimin either inhibits complex I or decreases mitochondrial membrane potential (which inevitably increases mitochondrial respiration in the absence of a respiratory chain inhibitor). However, Imeglimin did not either inhibit complex I activity (Table 1) or stimulate mitochondrial respiration (Table 2). We therefore propose that Imeglimin unconventionally affects complex I functioning, inhibiting the ROS production driven by a reverse electron transfer (Table 3) without inhibiting complex I activity or forward respiratory flux.

In the present study, we have also observed that Imeglimin prevented PTP opening and subsequent HMEC-1 death in a concentration- and time-dependent manner. Indeed, the effect obtained after 4 h using high concentration was reached using low (clinical range) concentration after longer (24 h) incubation time (Figures 1a–c), and that this response was still present when the cells were permeabilized after Imeglimin exposure, indicating the persistence of a putative mitochondrial change (Figure 3). In that respect, the case of Metformin is interesting to reconsider. Although it has been reported that millimolar range concentrations of Metformin directly inhibit complex I,^37–40^ much lower concentrations (micromolar range) are required to obtain the same result in intact cells.^19,27,41^ Note also that Metformin remains a mild inhibitor of complex I even at saturating concentration in shortly incubated HMEC-1 (~30 min), yielding a similar protection upon cell viability than after long-term exposure.^19^ On the other hand, it has recently been shown that 2 weeks of oral gavage of rats with Metformin led to complex I inhibition in skeletal muscle^42^ despite Metformin blood concentration is known to remain in the micromolar range in this condition. To explain this discrepancy, it has been proposed that Metformin slowly accumulates inside mitochondria, driven by the mitochondrial membrane potential.^38,40^ Although theoretically plausible, Metformin pharmacokinetic studies are not consistent with a huge (millimolar range) accumulation of Metformin in organs.^43,44^ It was thus concluded that although a direct effect of Metformin on complex I is possible, it seems to be considerably facilitated in intact cells regardless of the exact cellular mechanisms involved in this regulation.^41^ As the effect of Imeglimin on HMEC-1 also requires a lot of time, this suggests that Imeglimin slowly accumulates into the cell due to the lack or deficit of transporters, or that it indirectly affects complex I through an intricate signaling mechanism which remains to be explored in more detail.

Through PTP inhibition, the antiapoptotic effects of Imeglimin on endothelial cells suggest that it may have beneficial effects for diabetes-associated angiopathy. In addition, we could also explain, at least in part, the protective role of Imeglimin on hyperglycemia-induced cell death as the consequence of lower mitochondrial ROS production. If the contribution of reverse flux-related ROS generation under normal conditions remains still questionable, it seems however to be relevant in a pathological context. Indeed, a recently published study^45^ showed that ischemic accumulation of succinate was responsible for mitochondrial ROS production by reverse electron transfer at respiratory complex I during reperfusion. Studies in HFHSD mice have also demonstrated that Imeglimin decreases ROS production specifically in the presence of succinate.^6^ In the present study, we have confirmed that Imeglimin decreases ROS production, and that it does so by decreasing reverse electron transport at mitochondrial complex I. Further investigations are however necessary to conclude about the potential benefit of Imeglimin on the cardiovascular system.

## Conclusion

Imeglimin prevents oxidative stress- and hyperglycemia-induced cell death in HMEC-1 cells through inhibition of mitochondrial permeability transition, but without inhibiting complex I-driven mitochondrial respiration nor affecting both redox and phosphate potentials. Moreover, it has been shown that Imeglimin is able to decrease specifically ROS generation by reverse electron transport at complex I. These unique properties mean that, in addition to its antidiabetic effects, Imeglimin may help to prevent micro- and macrovascular complications in type 2 diabetes.

## Materials and Methods

### Cell culture conditions

The immortalized human dermal microvascular endothelial cell line HMEC-1^46^ was a kind gift from JJ Feige (CEA, Grenoble, France). The cells were grown to confluence in MCDB-131 culture medium supplemented with 15% heat-inactivated fetal bovine serum (FBS), 2 mM L-glutamine, 50 UI/ml penicillin, 50 *μ*g/ml streptomycin, 10 ng/ml epidermal growth factor and 1 *μ*g/ml hydrocortisone, and maintained in a humidified atmosphere (5% CO_2_) at 37 °C. Cells were trypsinized, then harvested by centrifugation at 1000 r.p.m. for 10 min.

### Quantification of endothelial cell death

HMEC-1 preincubated with or without Imeglimin (10 mM for 4 h or 100 *μ*M for 24 h) or with CsA (1 *μ*M for 30 min) were washed with PBS before subsequent exposure to 0.5 mM tBH in FBS-free culture medium for 45 min. Cells were then washed with PBS and incubated at 37 °C for 24 h in a complete MCDB medium. Alternatively, cells were exposed to 5.5 mM glucose (control cells) or to 33 mM glucose for 48 h. Cytotoxicity was evaluated with a double-stain system using the Annexin V-Fluoprobes 488 kit combined with propidium iodide. Data acquisition was carried out using a FACScan flow cytometer equipped with a 15 mW argon ion laser tuned at 488 nm.

### Immunohistochemistry of cytochrome *c*

For the visualization of cytochrome *c* by immunohistochemistry, cells were fixed in 3.7% paraformaldehyde/PBS for 20 min, permeabilized in 0.2% Triton X-100 for 5 min and blocked in 2% bovine serum albumin/PBS for 1 h. Cells were then incubated with a monoclonal anti-cytochrome *c* (clone 6H2.B4) for 2 h and exposed to Oregon green-labeled fluorescent secondary antibody in the dark at room temperature for 1 h. After final washes, 0.2 M Tris-HCl pH 7.8, 90% glycerol and 2.3% 1,4-diazobicyclo-[2.2.2]-octane (DABCO, anti-fading agent) was applied to cell preparations. Imaging was performed by confocal microscopy using a ×63/1.20 Plan Apo water immersion objective. Laser excitation was 488 nm, with a fluorescence emission adjusted with acousto optical beam splitter (AOBS) at 515–535 nm. Eight fields, randomly chosen with phase-contrast microscopy and containing about 10–20 cells each, were scanned by glass coverslips.

### Determination of mitochondrial permeability transition in permeabilized cells

PTP opening was evaluated *in vitro* by measuring the CRC of digitonin-permeabilized HMEC-1 cells. Cells were permeabilized immediately before use by incubation under stirring at 25 °C in a medium containing 250 mM sucrose, 10 mM Tris-MOPS (3-(*N*-morpholino)propanesulfonic acid), 1 mM Pi (inorganic phosphate)-Tris, 50 *μ*g/ml digitonin, and either 5 mM succinate or 5 mM/2.5 mM glutamate/malate (pH 7.35). The measurement of extra-mitochondrial calcium concentration was carried out fluorimetrically at 25 °C with a PTI Quantamaster spectrofluorometer equipped with magnetic stirring and thermostatic control in the presence of 0.25 *μ*M calcium green 5 N (excitation and emission wavelengths were set at 506 and 532 nm, respectively). Calcium loading was performed by repetitive additions of 12.5 *μ*M calcium until PTP opening occurred.

### Determination of mitochondrial permeability transition in intact cells

PTP opening was also observed *in vivo* using calcein/cobalt staining in living cells.^47^ HMEC-1 cells grown for 48 h on 22 mm diameter glass coverslips were exposed for 15 min at 37 °C to PBS medium supplemented with 5 mM glucose, 0.35 mM pyruvate, 1 mM CoCl_2_ and 1 *μ*M calcein-aceto-methoxyl ester. After loading, cells were washed free of calcein and CoCl_2_ and further incubated for 20 min at 37 °C in PBS/glucose/pyruvate medium. Cell imaging was performed with a LEICA TCS SP2 inverted laser confocal microscope, using a ×63/1.20 Plan Apo water immersion objective. Laser excitation was 488 nm, with a fluorescence emission adjusted with AOBS at 506–541 nm. Cell images were collected every minute with a constant exposure time.

### Assay of isolated respiratory chain complex I

Confluent monolayers of HMEC-1 cells were incubated in the absence or presence of Imeglimin (10 mM for 4 h or 100 *μ*M for 24 h). Cells were harvested, permeabilized in a digitonin-containing cold buffer and then spun down (10 000 r.p.m. for 10 min) to eliminate cytosolic contaminating enzyme activities. Permeabilized cells were placed in 800 *μ*l H_2_O in a stirred glass cuvette for 2 min at 30 °C to break mitochondrial membranes by hypotonic shock. Next, 200 *μ*l Tris solution (50 mM, pH 8.0) containing 50 *μ*M NADH was added for 1 min, and the reaction started by adding 50 *μ*M decylubiquinone as a final electron acceptor. NADH oxidation rate was measured fluorimetrically (excitation–emission, 340–460 nm). The rotenone-sensitive complex I activity was obtained after subtraction of the remaining signal in the presence of 10 *μ*M rotenone.

### Measurement of oxygen consumption rate in intact endothelial cells

After preincubation in MCDB-131 medium with or without Imeglimin or Metformin, intact HMEC-1 cells (1.5×10^7^ cells/ml) were placed at 37 °C in an oxygraph vessel equipped with a Clark oxygen electrode and filled up with MCDB-131 culture medium devoid of any supplements. The rate of oxygen consumption (*J*O_2_) was measured, then 2 *μ*g/ml oligomycin (in order to inhibit ATP synthesis) and 125 *μ*M DNP (in order to uncouple mitochondria) were successively added to the incubation medium.

### Assessment of energy metabolism

Cells were lysed by adding perchloric acid, which destroyed the enzymes but not the metabolites. After centrifugation at 12 000 r.p.m. for 5 min and neutralization of the supernatant with KOH (2 M)/MOPS (0.3 M), lactate and pyruvate were enzymatically measured while adenine nucleotides were measured by HPLC as previously described.^48^ The lactate/pyruvate ratio, which is proportional to the cytosolic NADH/NAD^+^ ratio, was taken as an index of the cytosolic redox potential.

### Detection of H_2_O_2_ production

The rate of H_2_O_2_ formation in permeabilized cells was measured fluorimetrically using amplex red (excitation–emission, 560–583 nm) in the presence of horseradish peroxidase. 2.5×10^7^ permeabilized cells were incubated at 30 °C in a medium containing 125 mM KCl, 20 mM Tris-HCl, 1 mM EGTA, 2.5 mM Pi-Tris (pH 7.35) and 10 *μ*M oligomycin. H_2_O_2_ production was initiated with glutamate/malate or succinate as respiratory substrates, then 5 *μ*M rotenone and 0.25 *μ*M antimycin A were sequentially added to measure the maximum rate of H_2_O_2_ production of complexes I and I+III, respectively.

### Statistical analysis

Data are presented as means+S.E.M. Statistical significance of differences was analyzed using the paired Student’s *t*-test.

## Figures and Tables

**Table 1:** Effect of Imeglimin on respiratory chain complex I activity in HMEC-1 cells

	*Control*	*Imeglimin* *100* *μM* – *24* *h*	*Imeglimin* *10 mM* – *4 h*
Total activity *J* NADH (nmol NADH/min/10^7^ cells)	1.43±0.034	1.44±0.042	1.39±0.049
After addition of rotenone (nmol NADH/min/10^7^ cells)	0.39±0.035	0.38±0.049	0.39±0.043
Rotenone-sensitive Complex I activity (nmol NADH/min/10^7^cells)	1.035±0.027	1.056±0.022	0.99±0.033

HMEC-1 cells incubated in the absence or presence of either 100 *μ*M Imeglimin for 24 h or 10 mM Imeglimin for 4 h were first permeabilized and then submitted to a hypotonic shock as described in the Material and Methods section. NADH oxidation rate was measured fluorimetrically in the presence of decylubiquinone before and after addition of rotenone. Results are means±S.E.M. of four independent experiments.

**Table 2:** Comparative effects of Imeglimin and Metformin on cellular oxygen consumption (*J*O_2_) and energy metabolism

	*Control*	*Imeglimin*	*Metformin*
*JO_2_ (Natom O/min/mg protein)*			
No addition	4.9±0.14	4.6±0.22	3.1±0.14^a^
+ Oligomycin	1.6±0.04	1.5±0.03	1.3±0.07
+ DNP	9.1±0.76	9.2±0.41	6.1±0.51^a^
			
*J* lactate (*μ*mol NADH/30 min/mg protein)	3.65±0.27	3.81±0.30	4.90±0.29^a^
Lactate/pyruvate ratio	1.59±0.11	1.65±0.13	2.23±0.12^a^
ATP (nmol/mg prot)	83.9±4.37	81.6±1.7	69.7±4.95^a^
ADP (nmol/mg prot)	17.28±3.07	17.38±1.38	21.02±2.78
ATP/ADP	5.09±0.70	4.8±0.25	3.37±0.23^a^

HMEC-1 cells incubated in the presence of either 10 mM Imeglimin for 4 h or 10 mM Metformin for 30 min. *J*O_2_ was measured before and after the successive additions of oligomycin and DNP. In parallel experiment, the production of lactate and the lactate-to-pyruvate ratio, as well as the content of ATP and ADP, was determined as described in the Material and Methods section. Results are means±S.E.M. of four independent experiments.

a *P*<0.05 *versus* control cells, paired Student’s *t* -test.

**Table 3:** Effect of Imeglimin on mitochondrial H_2_O_2_ production

	*H_2_O_2_* (*pmol/min/10^7^ cells*)					
	*Glutamate/malate*		*Succinate*		*Glutamate/malate+succinate*	
	*Control*	*Imeglimin*	*Control*	*Imeglimin*	*Control*	*Imeglimin*
Basal	32.3±2.4	28.4±2.2	25.7±3.0	14.5±1.3^a^	22.3±1.5	15.7±1.4^a^
+Rotenone	38.3±3.2	37.5±1.5	10.7±1.8	11.4±2.7	7.8±0.9	6.9±1.1
+Antimycin A	51.0±4.8	51.4±8.7	20.1±3.3	19.1±3.2	20.5±1.4	18.6±1.3

HMEC-1 cells incubated in the absence or presence of 100 *μ*M Imeglimin for 24 h were digitonin-permeabilized in a KCl respiratory medium supplemented with Amplex Red/HRP. Mitochondria were energized with glutamate/malate, succinate or both substrates (glutamate/malate plus succinate) in order to measure the basal H_2_O_2_ production, then 5 *μ*M rotenone and 0.25 *μ*M antimycin A were added. Results are mean±S.E.M. of five different experiments.

a *P*<0.05 *versus* control, paired Student’s *t*-test.

## Abbreviations

PTP: permeability transition pore
AIF: apoptosis inducing factor
HMEC-1: human endothelial cells
CsA: Cyclosporine A
ROS: reactive oxygen species
tBH: tert-butyl hydroperoxide
NAC: N-acetyl cysteine
FBS: fetal bovine serum
BSA: bovine serum albumin
AOBS: acousto optical beam splitter
CRC: calcium retention capacity
MOPS: 3-[N-morpholino]propanesulfonic acid
*J*O_2_: rate of oxygen consumption
DNP: dinitrophenol
PCA: perchloric acid.
